# Early Discharge for Anterior Cervical Fusion Surgery: Prediction of Readmission and Special Considerations for Older Adults

**DOI:** 10.3390/ijerph16040641

**Published:** 2019-02-21

**Authors:** Yu-Chun Chen, Jau-Ching Wu, Hsuan-Kan Chang, Wen-Cheng Huang

**Affiliations:** 1 Department of Family Medicine, School of Medicine, National Yang-Ming University, Taipei 11221, Taiwan; 2 Department of Family Medicine, Taipei Veterans’ General Hospital, Taipei 11217, Taiwan; 3 Institute of Hospital and Health Care Administration, National Yang-Ming University, Taipei 11221, Taiwan; 4 Department of Biomedical Engineering, School of Biomedical Science and Engineering, National Yang-Ming University, Taipei 11221, Taiwan; 5 Department of Neurosurgery, School of Medicine, National Yang-Ming University, Taipei 11221, Taiwan; jauching@gmail.com (J.-C.W.); hsuankanchang@gmail.com (H.-K.C.); wchuang@vghtpe.gov.tw (W.-C.H.); 6 Department of Neurosurgery, Neurological Institute, Taipei Veterans’ General Hospital, Taipei 11217, Taiwan

**Keywords:** anterior cervical discectomy and fusion (ACDF), early discharge (ED), incidence rates, readmission, reoperation

## Abstract

Anterior cervical discectomy and fusion (ACDF) is the standard surgical management for disc herniation and spondylosis worldwide and reportedly performed with short hospitalization and early discharge (ED). However, it is unknown if ED improves the outcomes of ACDF including among older adults. This cohort study included patients who underwent ACDF surgery in Taiwan over two years analyzed in two groups: the ED group (discharged within 48 hours), and the comparison group (hospitalized for more than 48 h). Both groups were followed-up for at least 180 days. Pre- and post-operative comorbidities, re-admissions and re-operations were analyzed using a multivariate cox-regression model, with bootstrapping, and Kaplan–Meier analysis. Among 5565 ACDF patients, the ED group (*n* = 405) had a higher chance (crude and adjusted hazard ratio = 2.33 and 2.39, both *p *< 0.001) of re-admission than the comparison group (*n* = 5160). The ED group had an insignificant trend toward more re-admissions for spinal problems and re-operations within 180 days. In the ED group, older age (≥60) and hypertension were predictive of re-admission. For ACDF surgery, the ED group had higher rates of re-admission within 180 days of post-op, suggesting that the current approach to ED requires modification or more cautious selection criteria be adopted, particularly for older adults.

## 1. Introduction

Since 1958, when Cloward invented his approach for anterior cervical discectomy and fusion (ACDF), this surgery has become widely accepted for the management of medically refractory cervical myelopathy, as well as for radiculopathy [1,2,3,4,5]. For patients who have received one- or two-level ACDF, there have been very high rates of satisfaction and low incidences of complications [6,7,8]. Multi-level ACDF also has gained popularity within recent decades owing to the advances made in biologics and instrumentation [9,10,11]. Thus, ACDF has become one of the most commonly performed surgeries in regular neurosurgical practice and has frequently resulted in excellent clinical outcomes.

There has been an emerging adaptation of early discharge (ED) in many fields of surgery, beginning with enhanced recovery after surgery (ERAS) used for colorectal surgery [12,13,14]. Among the subspecialties of neurosurgery, spinal surgery has a great potential to take advantage of ED. The surgical approach for anterior cervical spinal fusion itself involves little disruption of local musculatures or other systemic intervention. Although general anesthesia and hospitalization is usually required for patients who undergo ACDF surgery, efforts have been made by experts in spine care to expedite the treatment algorithm and reduce the length of stay needed for ACDF [15,16]. In the United States, many ACDF operations are performed in ambulatory care settings, and patients can go home the same day after surgery [11,17,18]. However, in Asia, where patients are more used to longer lengths of stay in hospitals, ACDF patients have averaged several days of hospitalization. There has not been a large cohort study to investigate the effects of ED following ACDF and the length of hospitalization on clinical outcomes.

The current study aimed to compare the differences in clinical outcomes, including rates of re-admission and re-operation, after ACDF, taking into account the various lengths of hospitalization. More than 6200 ACDF patients with complete follow-ups were compared in order to investigate the effects of ED. Due to the comprehensive coverage of the national health insurance policy, these ACDF patients were tracked for any subsequent medical care services. To date, this was the first and largest cohort study to address the effects of ED on post-ACDF outcomes.

## 2. Materials and Methods 

### 2.1. Data Source and Ethical Concerns

This study used Taiwan’s open claim database, the National Health Insurance Research Database (NHIRD). Taiwan’s Government launched a National Health Insurance (NHI) program in 1995, aimed at providing unrestricted access to medical care and universal health insurance for all residents in Taiwan. To date, the NHI program has comprehensively covered 99% of the Taiwanese population and has contracted with 97% of the providers of healthcare services in Taiwan. After cross-checking for validity, de-identification, and anonymization, the claim data of the NHI program have been released publicly for research purposes. The NHIRD contains comprehensive information on the insured subjects, including gender, date of birth, dates of clinical visits (both preventive services and emergent visits) and hospitalization, the International Classification of Diseases (Ninth Revision) Clinical Modification (ICD-9-CM) codes of diagnoses, ICD codes of surgical procedures, etc.

This study’s protocol was approved by the Institutional Review Board of Taipei Veterans General Hospital (IRB# 2018-09-0006CC). The Institutional Review Board waived the requirement for written informed consent from each of the patients involved since all identifying personal information in the NHIRD was encrypted.

### 2.2. Identification of Study Cohort, and Hospitalization for ACDF

**Figure 1:**
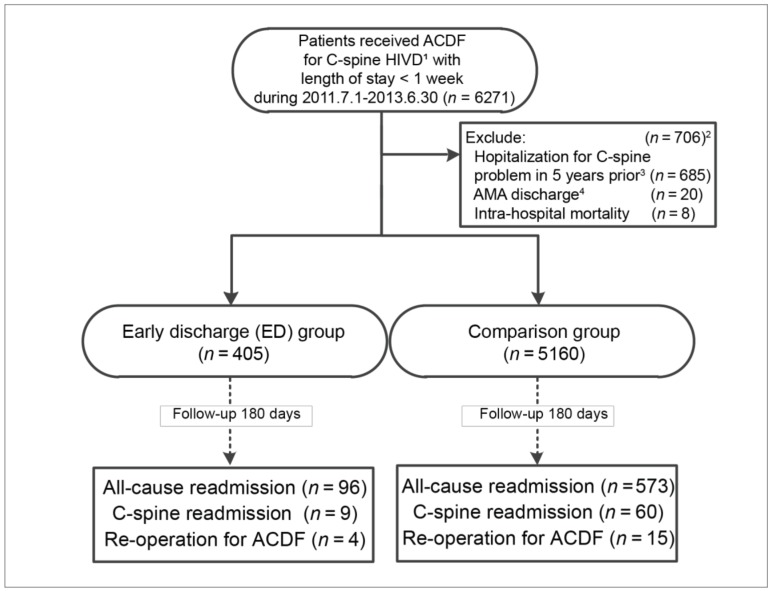
Flowchart of data processing for outcomes of a cohort of anterior cervical discectomy and fusion (ACDF), the early discharge (ED) group versus the comparison group, in Taiwan, 2011–2013 (*n* = 6,271). ^1^ HIVD, herniation of inter-vertebral disc. ^2^ The total number for all of the criteria exceeded 706 because 7 patients met multiple criteria. ^3^ Patients were tracked back for 5 years, thus, only cervical spine problems in the 5 years prior to the index surgery were excluded. ^4^ AMA discharge, discharge against medical advice (mostly for non-medical reasons).

This was a population-based retrospective cohort study to focus on patients who underwent ACDF, the standard surgical procedure for medically refractory cervical disc diseases that cause radiculopathy or myelopathy. To focus on patients with similar conditions to spinal problems, we only included patients in our eligible cohort whose length of stay (LOS) was less than or equal to 6 days, that is, the median of all patients who received ACDF during the study period in Taiwan. As a result, we enrolled all patients who had been hospitalized for cervical intervertebral disc disease (ICD-9-CM: 722.0, 722.4 and 722.7) and who had received cervical discectomy (ICD-9 operative procedure code: 80.51) in combination with spinal fusion (ICD-9 operative procedure code: 81.00, 81.02, 81.30, and 81.32) with a LOS of less than or equal to 6 days between July 1, 2011 and June 30, 2013 (which is a similar code definition as in our previous work [19]). Traced back for at least 5 years, patients who had previous ACDF surgery or hospitalization for related problems, discharge against medical advice (AMA discharge, mostly for non-medical reasons), or intra-hospital mortality were excluded from the analysis. (Figure 1).

For each admission in our study cohort, we designated the first day for hospitalization as the index date for the ACDF surgery, and the length of admission for hospitalization was calculated. We further limited our study cohort to the length of stay between one and six days to reduce outliers (e.g., complicated surgery) and to make our study cohort more homogenous and comparable.

### 2.3. ED Group vs. Comparison Group

All patients were assigned to either the ED group or the comparison group according to the length of stay in hospital. Patients in eastern Asian countries typically have a longer length of stay for any surgery requiring hospitalization, because of cultural reasons, welfare issues, and the payment system. Therefore, the median length of stay for routine elective ACDF surgery would be approximately 6 days, including pre-operative preparation before the surgery. However, since 2010, Taiwan’s Bureau of National Health Insurance (BNHI) gradually implemented the Diagnosis-Related Group (DRG) payment system for 1062 major surgeries including ACDF to give medical facilities greater incentive to reduce the medical resource expenditure (BNHI, 2013). Since then, spine surgeons have begun to adopt the strategy of ED to increase the efficiency of health care services and to reduce the length of stay for ACDF. As a result, it is a common practice for patients admitted to hospital to have ACDF surgery on the same day or the next day. The current study used the first day of admission as the index date of ACDF, as the exact operation time was not available.

Most patients who undergo ACDF surgery have an uneventful course of hospitalization. Since ACDF is the standard of care as a surgical option for cervical disc diseases and spondylosis, it has extremely high rates of success and low rates of complications. Most patients are discharged within days post-operation. Usually patients can be discharged when there is little concern of neurological, respiratory, or digestion issues. 

To compare the outcome of ED to conventional procedures, we assigned patients who were hospitalized, successfully operated on and discharged in less than 48 h as the ED group; patients who had a length of hospitalization longer than 48 hours, but less than 6 days, were assigned as the comparison group. A sensitivity test was performed to examine the effect of a change in outcome if the definition of ED changed (Table A1).

### 2.4. Follow-Up Outcomes and Risk Factors

The most clinically related outcomes of ACDF, a common neurosurgical procedure with extremely high patient satisfaction, would be re-admissions and re-operations. Since the NHIRD uniquely provides a very comprehensive follow-up of the entire cohort, all patients in the study were followed up on for 180 days after discharge from hospital. Owing to the comprehensiveness of the NHIRD, we were able to track every subsequent re-admission in Taiwan with a very minimal loss of follow-up. Following re-admission and subsequent discharge, we used the discharge codes as the reason for the re-admission and followed up on the first occurrence of each of three re-admission categories: all-cause re-admission, cervical spine-related re-admission (ICD-9-CM: 722.0, 722.4, and 722.7), and re-operations (ICD-9 operative procedure code: 80.51 in combination with 81.00, 81.02, 81.30, and 81.32). Thus, any re-admissions and re-operations within 180 days after the indexed surgery would be tracked, even if they were in different institutes or physically distant, because the universal coverage by the government supported monopolistic health insurance scheme enabled this. Also, because all medical care providers are contracted with the BNHI, which allows all patients unrestricted access, any post-operative events would very likely be captured by the NHIRD. Due to the rigorously monitored billing processes, the re-admissions and re-operations of these patients would have little chance of loss to follow-up.

All reasons for re-hospitalization, including specific re-hospitalizations for cervical spinal problems, and re-operations, were analyzed at 30, 60, and 180 days for comparison between the ED group and the comparison group. Furthermore, all patients were traced back for 5 years in the NHIRD for any previous out-patient clinic visit or hospitalization, to ameliorate the chances of confounding issues caused by previous cervical spinal diseases. All suspected spinal conditions, including a history of cervical spinal surgery, were thus excluded.

To evaluate the effects of ED on re-admissions and re-operations, we included potential risk factors such as age, sex and other identifiable factors in a multivariate analysis. We categorized and included the most prevalent co-morbidities according to Elixhauser’s co-morbidity model. Patients’ co-morbidities were determined by the presence of either diagnostic codes in the outpatient records or discharge codes in the database within two years before the date of the index date [20,21]. A total of 8 kinds of medical co-morbidities of the highest prevalence rates (>3.5%) were included as the predictive factors. These medical co-morbidities included anemia, chronic peptic ulcer disease, chronic pulmonary diseases, depression, diabetes, hypertension, chronic hepatic diseases, and valvular heart diseases; together with age and gender, these were analyzed for the prediction of re-admission.

### 2.5. Outcome Analysis and Sub-Group Analysis for Older Adults

To analyze the reasons for re-admission, we categorized the main discharge codes by using the Clinical Classification System (CCS) 2015 of the U.S. Agency for Healthcare Research and Quality (http://www.hcup-us.ahrq.gov/toolssoftware/ccs/ccs.jsp). The reasons for re-admission were thus grouped into one of 15 clinically meaningful categories, and the incidence rates (IR) of re-admission were calculated and compared between the ED and comparison groups.

To further quantify the outcomes of older adults, a sub-group analysis was conducted for patients aged ≥60 years. The effects of ED on re-admission, re-admission for C-spine problems, and a second ACDF operation were examined.

### 2.6. Statistical Analysis

All the data were linked using the SQL server 2017 (Microsoft Corp, Redmond, WA, USA) and analyzed by Stata software (Stata Corp, College Station, TX, USA). The Kaplan–Meier method and a log-rank test were used to estimate and compare cumulative re-admission and re-operation rates among different groups. An adjusted hazard ratio (aHR) and a 95% confidence interval (95% CI) for re-admission were estimated using a Cox proportional regression model. For the relatively rare outcomes, such as re-admission for cervical spine-related problems and re-operations, we used a bootstrap method with 1000 repeat re-sampling to obtain a less-biased estimation of aHR, with a standard error (SE) of adjusted hazard ratio and *p*-value reported instead of the conventional 95% CI. A risk analysis was performed for the ED group, and a Cox regression model was used to quantify the influence of re-admission of each risk factor by controlling other risk factors. A two-tailed level of 0.05 was considered statistically significant.

## 3. Results

A total of 6271 patients who underwent ACDF surgery between July 2011 and June 2013 were identified in the NHIRD (Figure 1). Among them, 706 were excluded from analysis because of previous ACDF surgery or hospitalization for related problems (*n* = 685), for having been discharged from hospitalization without surgery (*n* = 20), or for mortality (*n* = 8); (the total number exceeded 706, because 7 patients met multiple exclusion criteria). The analysis was conducted for a total of 5565 patients.

There were 405 patients in the ED group who were hospitalized for less than 48 h for pre-operation management, surgery, and post-operative management. For the comparison group, there were 5160 patients, who were hospitalized for more than 48 h (up to 6 days). All patients were then followed up on for the next 180 days after the indexed surgery (i.e., ACDF; see Figure 1).

### 3.1. Preoperative Demographics

The comparison group and the ED group were similar in pre-operative conditions, including sex, age, and medical co-morbidities. The gender distribution was not different between the comparison group and the ED group (Female: Male, 47.7%:52.3% vs. 45.7%:54.3%, *p* = 0.443). The mean ages between the two groups were also very similar (55.1 years vs. 54.5 years, comparison group vs. ED group, *p* = 0.327). Medical co-morbidities, including anemia, chronic peptic ulcer disease, chronic pulmonary disease, depression, diabetes, hypertension, liver diseases, and valvular heart diseases were very similar between the comparison and ED groups (Table 1).

### 3.2. Rates of Re-Admission and Re-Operation

The comparison group and the ED group were very different in re-admissions and re-operations. The two groups were compared for rates of all-cause re-admissions, cervical spine related re-admission, and re-operations within 30, 60, and 180 days.

**Figure A1:**
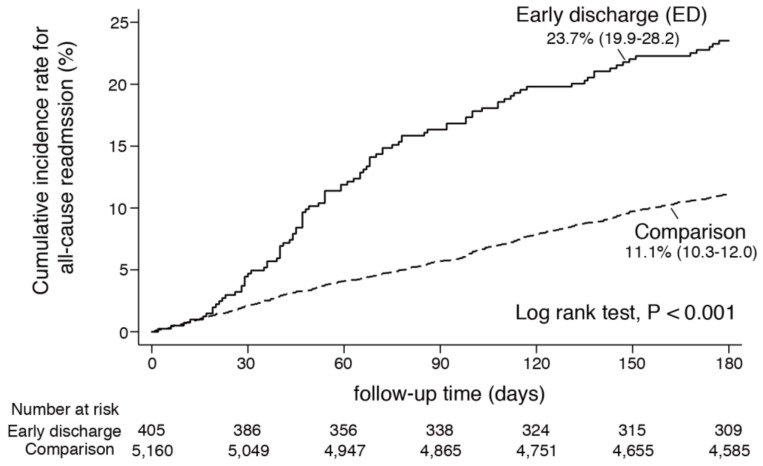
Cumulative incidences of all-cause re-admissions during the 180-day follow-up of both the comparison and early discharge (ED) groups after the indexed ACDF surgery, 2011–2013 (*n* = 5565).

The ED group had a higher rate of all-cause re-admission than the comparison group within 30, 60 and 180 days (4.9% vs. 2.2%, 12.1% vs. 4.1%, and 23.7% vs. 11.1%, all *p* < 0.001; see Table 1). Also, the ED group had a higher rate of cervical spine-related re-admission within 30, 60, and 180 days (1.5% vs. 0.2%, 1.5% vs. 0.5%, and 2.2% vs. 1.2%, *p* < 0.001, 0.01, and 0.001). Furthermore, the ED group had a higher rate of re-operations within 30, 60, and 180 days (0.7% vs. 0.03%, 0.7% vs. 0.1%, and 1.0% vs. 0.3%, *p* < 0.001, 0.01, and 0.05). The Kaplan–Meier survival analysis also demonstrated significantly higher accumulative incidences of re-admission to 180 days after the indexed ACDF surgery (23.7% vs. 11.1%, log-rank test, *p* < 0.001; see Figure A1). 

The re-operation (ACDF) rates were extremely low, which were 0.3% and 1.0% at 180 days post-operation for the comparison and ED groups, respectively. (Table 1) Other kinds of secondary surgery would be counted together with repeat ACDF in the cervical-spine related re-admissions, which were for the comparison and ED groups 1.2% and 2.2%, respectively, at 180 days post-operation. There were multiple possibilities that caused these re-operations, including wound infection, pseudarthrosis, adjacent segment disease, and implant failure. However, the exact cause and types of procedures were beyond the scope of the database used in the current study.

Adjustment of the demographics and medical co-morbidities (as that listed in Table 1) were made to mitigate the confounding factors for a comparison of the outcomes at 180 days. In summary, patients of the ED group were more likely to be re-admitted to a hospital for whatever reasons (all-cause re-admission crude HR = 2.33, and adjusted HR = 2.39, both *p* < 0.001) (Table 2). Although the incidence rates of cervical spine-related re-admissions and re-operations were higher in the ED group, they did not reach any significant levels.

### 3.3. Risk Analysis

**Figure 2:**
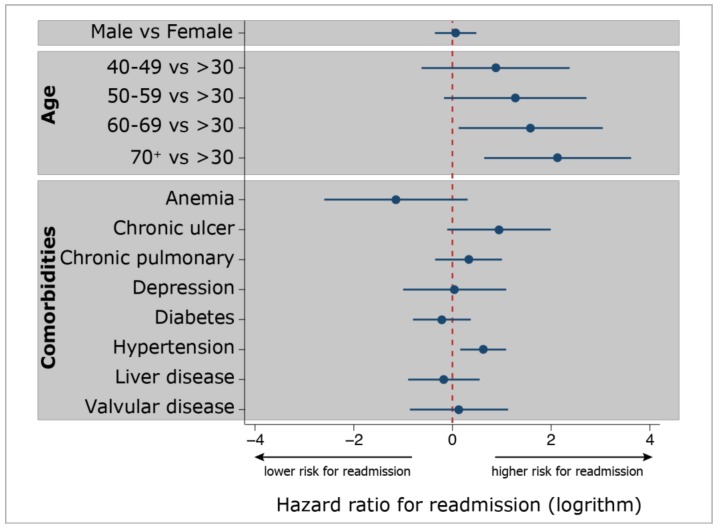
Adjusted hazard ratios for re-admission for the early discharge (ED) group after the ACDF surgery by potential risk factors (2011–2013, *n* = 405). A positive value of the logarithm of adjusted hazard ratio implies significant risks for re-admission.

Older age (more than 60 or 70 years) and chronic hypertension were the risk factors of re-admission after the indexed ACDF surgery for patients in the ED group. (Figure 2) The other variates, including sex, other age groups, and other medical co-morbidities were estimated for all-cause re-admission by 180 days. However, most of them did not reach significances.

### 3.4. Outcome Analysis

Patients were mostly re-admitted for reasons other than a C-spine problem. Although the re-admission rate (IR = 259.73 per 1000 person-years, 95% CI = 240.74–280.23) was high in the study cohort, only a small portion of them were hospitalized for a C-spine problem (IR = 24.6 per 1000 person-years, 95% CI = 19.4–31.3; see Table 2).

Diseases of the musculoskeletal system and connective tissue were the leading reasons (38% of total re-admissions) for re-admission after the index ACDF surgery, followed by injuries (13%) and diseases of the circulatory system (9%). The re-admission rate for diseases of the musculoskeletal system and connective tissue in the ED group (IR = 246.9 per 1000 person-years) was three times higher than that in the comparison group (IR = 79.1 per 1000 person-years, IRR = 3.12, *p* < 0.001; see Table 3).

### 3.5. Subgroup Analysis for Older Adults

Generally, older adults had a higher risk for re-admission following the index surgery than younger adults. The incidence rate for re-admission was higher for older adults in both the ED group (IR = 724.64 per 1000 person-years, 95% CI = 603.85–825.37) and comparison group (IR = 317.16 per 1000 person-years, 95% CI = 285.87–349.73) than younger adults in corresponding groups (younger adults, ED group, IR = 379.13 per 1000 person-years, 95% CI = 283.08–507.79; comparison group, IR = 185.82 per 1000 person-years, 95% CI = 166.03–207.96, both *p* < 0.001) (Table 4).

Similarly, older adults were mostly re-admitted for reasons other than a C-spine problem. Admission for C-spine problems (IR= 32.82 per 1000 person-years, 95% CI = 22.25–46.53) accounted for approximately 10% of re-admission rates in older adults (IR = 376.30 per 1000 person-years, 95% CI = 343.60–410.00), whereas re-admission rates for older adults in the ED group remained two times higher than that in the comparison group (*p* < 0.001) (Table 4).

## 4. Discussion

This study analyzed, on a national scale, the outcomes of a large cohort of patients who underwent ACDF surgery. There were 405 patients in the ED group, who were hospitalized for 48 h for all pre-operative examinations, surgery, and post-operation recovery. On the other hand, 5160 patients who received ACDF surgery without the fast-tracked management of ED and who were typically hospitalized for a total of 3–6 days were analyzed as the comparison group. All patients were followed up on for 180 days post-operation, and for all events of re-admission and re-operation.

The patients of the ED group had a higher incidence of all-cause re-admission (adjusted HR = 2.39) than those of the comparison group. When looking specifically into the details, re-admissions related to cervical spinal problems (which could be directly connected to the ACDF surgery) and those who required re-operations were not statistically different between the two groups. In other words, the ED group had more re-admissions caused by other medical conditions. Therefore, in this cohort of ACDF patients, ED yielded more re-admissions, although these were likely not caused by the surgery itself.

Patients who were older than 60 years and those who had hypertension had a higher incidence of such re-hospitalizations. This was the first study, with the largest patient number and highest follow-up rate, to demonstrate that ED following ACDF surgery must be cautiously applied for older adults. More longitudinal and multi-disciplinary efforts should be incorporated into ED among older adults who undergo ACDF surgery. Otherwise, ED could cause higher re-admission rates within 6 months post-operation.

More longitudinal and multi-disciplinary efforts in addition to ED might be required to enhance the outcomes following ACDF surgery, especially for older adults. Validated evidence and experience has largely supported the application of the strategical management for ED programs in large-volume orthopedic surgery. For example, Aasvang et al. performed a narrative review on fast-track surgery in patients discharged 1–3 days post-operatively and concluded that ED is routinely applicable for total hip and knee replacement with unchanged re-admission [22]. There was little research specifically addressing the utilization of the ED program in cervical spine surgery. Venkata et al. retrospectively reviewed 50 cases that underwent the ED program for cervical spinal surgery, with one-year follow-up. Post-operatively, 2.1% of the cases presented to emergency departments, and re-admissions accounted for 2.5% of the series within 30 days after surgery. In the series, six patients required a second operation [23]. The current study suggested that ED alone is not enough for ACDF patients. A longitudinal ED program (such as ERAS) of concerted institutional-level multi-disciplinary efforts would reduce the burden of surgery through a combination of changes in practice and management [12,13,14].

However, if we consider the setting of ambulatory surgery centers (ASC) as a practice of the ED program, published articles are more supportive of the application in cervical spine surgery. In ASCs, the complication rates of ACDF ranged from 0% to 5.2%. Rates of hospital transfer ranged from 0% to 6%, and rates of re-admission ranged from 0% to 2.2% [15,17,24,25]. Retrospective cohort studies usually showed low complication and re-admission rates. However, most of the retrospective series evaluated short-term morbidities within 30 days [26,27]. Comparison with patients undergoing a conventional discharge plan and longer-term data is lacking. Our study included both early and non-early discharged patients, and the follow-up period was extended beyond 1 month after surgery. The present study was the first to demonstrate unfavorable results of ED for ACDF when follow-up periods were longer and up to 180 days, in contrast to those in which follow-up was relatively short (<30 days).

Although ACDF is such a common neurosurgical procedure that frequently yields high patient satisfaction, the re-admissions within 6 months post-operation should not be overlooked. In the current study, the all-cause re-admission rates at 180 days post-operation were quite substantial, at 11.1% and 23.7% for the comparison and the ED groups, respectively. The re-admission rates were even higher in the older group. The present study warrants a modification of the peri-operative management to facilitate discharge, especially for older patients. Furthermore, strategies to promote global health conditions for older patients are needed to mitigate the high chance of re-admission within 6 months. The follow-up for these older patients might require a thorough consideration of rehabilitation, medical comorbidities, and other post-acute management. According to the current study, a post-operative management program tailored for each patient for a minimal 3 months (the median time demonstrated in Table 4) might be helpful in reducing re-admission numbers.

There were limitations in the current study. The NHIRD provided no details of each patient’s operative note. Therefore, the cohort of patients might have undergone different kinds of ACDF surgery, including one or multi-level fusion, instrumented or non-instrumented fusion, and used various kinds of grafting materials and biologics. All the above variants of ACDF could have affected the length of stay and chances of subsequent re-admission or re-operations. Furthermore, there was a lack of uniform protocol to achieve ED in the current cohort. Because it was a multi-center study, each surgeon or institute could have adopted various kinds of ED management. For instance, some could have focused more on the preparation for surgery, while others may have emphasized more the post-operative care, and even some may have involved specialized anesthesia or surgical techniques. These multiple factors could also play some role in the effectiveness of the ED strategy. Therefore, the 30, 60, and 180-day re-admission and re-operation rates could be influenced by the variables mentioned above. However, this study provided the best available evidence that ED could be applied for ACDF surgery safely in a multi-center, longitudinal cohort. The re-operations were unlikely related to ED and the re-admissions were likely caused by non-spinal medical conditions. Despite the variable protocols of ED that could have been included from multiple institutes, the study demonstrated that older age and cardiovascular disorders (e.g., hypertension) were the predictive risk factors of re-admission for ED patients of ACDF. All in all, this could be a valuable contribution to the literature, though it did not support the practice pattern of North America. 

## 5. Conclusions

In this cohort of ACDF patients, those who were discharged early had higher rates of re-admission within 180 days post-operation. Either the strategies to achieve ED in the cohort required modification and refinement, or the utilization of ED for ACDF patients’ needs to be more cautious and selective, especially amongst older adults who were at greater risk of re-admission.

## Figures and Tables

**Table 1:** Demographic characteristics, co-morbidities, and outcomes of the comparison and the early discharge (ED) groups, 2011–2013 (*n* = 5565).

Characteristics and Outcomes	Comparison Group		ED Group		*p*-Value
	*n* = 5160	(%)	*n* = 405	(%)	
**Gender**					0.443
Female Male	2459	(47.7)	185	(45.7)	
	2701	(52.3)	220	(54.3)	
Age, mean (SD)	55.1	(11.37)	54.5	(11.29)	0.327
**Co-morbidities**					
Anemia	217	(4.2)	14	(3.5)	0.467
Chronic peptic ulcer disease	186	(3.6)	10	(2.5)	0.233
Chronic pulmonary disease	602	(11.7)	46	(11.4)	0.852
Depression	268	(5.2)	16	(4.0)	0.274
Diabetes	996	(19.3)	74	(18.3)	0.612
Hypertension	1749	(33.9)	130	(32.1)	0.462
Liver disease	530	(10.3)	38	(9.4)	0.569
Valvular disease	20	(4.0)	13	(3.2)	0.436
**Outcomes**					
All-cause re-admission					
30-day re-admission	113	(2.2)	20	(4.9)	<0.001
60-day re-admission	214	(4.1)	49	(12.1)	<0.001
180-day re-admission	573	(11.1)	96	(23.7)	<0.001
**Cervical spine-related re-admission**					
30-day re-admission	12	(0.2)	6	(1.5)	<0.001
60-day re-admission	25	(0.5)	6	(1.5)	0.009
180-day re-admission	60	(1.2)	9	(2.2)	<0.001
**Re-operations**					
30-day re-operation	2	(0.0)	3	(0.7)	<0.001
60-day re-operation	6	(0.1)	3	(0.7)	0.003
180-day re-operation	15	(0.3)	4	(1.0)	0.021

**Table 2:** All-cause re-admissions, cervical spine-related re-admissions, and re-operations during 180 days of follow-up of both the comparison and early discharge (ED) groups after the index ACDF surgery, 2011–2013. (*n* = 5565).

Re-Admission during 180-Day Follow-Up	Comparison Group	ED Group
All-cause re-admissions Incidence of all-cause re-admissions		
(per 1000 person-years)	235.1	548.5
Number of occurrences	572	95
Observed person-years	2428.4	173.2
Crude hazard ratio (95% CI)	1.00	2.33 (1.86–2.90) ***^2^
Adjusted hazard ratio (95% CI) ^1^	1.00	2.39 (1.92–2.97) ***^2^
Cervical spine-related re-admissions Incidence of cervical-spine-related re-admissions (per 1000 person-years)	23.0	40.2
Number of occurrences	59	8
Observed person-years	2561.4	198.8
Crude hazard ratio (95% CI)	1.00	1.75 (0.72–3.67)
Adjusted hazard ratio (SE, *p*-value) ^3^	1.00	1.72 (SE = 2.13, *p*-value = 0.660)
Re-operations Incidence of re-operations		
(per 1000 person-years)	5.8	19.9
Number of occurrences	15	4
Observed person-years	2573.9	200.8
Crude hazard ratio (95% CI)	1.00	3.42 (0.83-10.73)
The adjusted hazard ratio (SE, *p*-value) ^3^	1.00	3.27 (SE = 18.80, *p*-value = 0.852)

^1^ Adjusted for age, gender, anemia, chronic peptic ulcer disease, chronic pulmonary disease, depression, diabetes, hypertension, liver disease, and valvular disease. ^2^ Significance level: ***, *p* < 0.001. ^3^ The adjusted hazard ratio was estimated based on a multivariate Cox regression model with 1000 repeats of bootstrap samples. Standard error (SE) of adjusted hazard ratio and *p*-value were reported instead of 95% CI A larger SE means a wider confidence interval and implies less chance that ED associates with the outcome (i.e., C-spine re-admission or re-operation for ACDF).

**Table 3:** Numbers, incidence rates (IR) and incidence rate ratios (IRR) by reasons for re-admission for early discharge and comparison groups after the index ACDF surgery, 2011–2013. (*n* = 5565).

Reasons for Re-Admission	Comparison Group *n* = 5160		ED Group *n* = 405		Incidence Rate Ratio (IRR) ^2^			
	*n*	IR ^1^	*n*	IR ^1^	IRR	(95% CI)	*p*-value	Sig. ^3^
**Musculoskeletal system & connective tissue**	204	79.1	50	246.9	3.12	(2.24–4.27)	<0.001	***
Injuries	74	28.7	14	69.1	2.41	(1.26–4.30)	0.006	**
Diseases of the circulatory system	55	21.3	5	24.7	1.16	0.36–2.87)	0.715	
Diseases of the respiratory system	38	14.7	1	4.9	0.34	(0.01–1.98)	0.266	
Neoplasms	38	14.7	3	14.8	1.01	(0.20–3.17)	0.931	
Diseases of the nervous system	36	14.0			−6.98	(−9.26–4.70) ^4^	0.033	*
Diseases of the digestive system	33	12.8	6	29.6	2.32	(0.79–5.60)	0.082	
Diseases of the genitourinary system	31	12.0	4	19.8	1.64	(0.42–4.65)	0.356	
Endocrine, metabolic, and immunity disorders	15	5.8	2	9.9	1.70	(0.19–7.30)	0.476	
Congenital anomalies	13	5.0	10	49.4	9.80	(3.85–24.19)	<0.001	***
Others	36	14.0	1	4.9	0.35	(0.01–2.10)	0.300	

^1^ Incidence rates (IR) were calculated by assuming each patient was followed-up for half the year and expressed in per thousand person-years. ^2^ Incidence rate ratio (IRR) and 95% confidence interval (95% CI) were point estimates. ^3^ Significance level: ***, *p* < 0.001; **, *p* < 0.01; *, *p* < 0.05 ^4^ Incidence rate difference (IRD) was expressed instead of IRR.

**Table 4:** Numbers, median time to re-admission and incidence rates (IR) stratified by outcomes, early discharge (ED) group and comparison group after the index ACDF surgery in older adults, 2011–2013. (*n* = 1827).

Outcomes in Older Adults (*n* = 1827)	n	Median Time to Re-Admission (days)		Incidence Rate (IR) (per 1000 Person-Years)		*p*-Value
		Days	(95% CI)	IR	(95% CI)	
**Any re-admission**						
Overall	318	80.0	(9.0–95.0)	376.30	(343.60–410.00)	
ED group	50	54.0	(19.0–151.0)	724.64	(603.85–825.37)	<0.001
Comparison group	268	87.5	(7.0–168.0)	317.16	(285.87–349.73)	
Re-admission for C-spine problem						
Overall	30	38.5	(1.0–148.0)	32.82	(22.25–46.53)	
ED group	5	26.0	(6.0–70.0)	72.46	(23.95–16.11)	0.082
Comparison group	25	40.0	(1.0–148.0)	29.59	(19.24–43.37)	
Re-admission for second ACDF						
Overall	6	46.5	(6.0–155.0)	6.56	(3.53–14.23)	
ED group	2	16.0	(6.0–26.0)	28.99	(3.53–100.82)	0.049
Comparison group	4	54.5	(40.0–155.0)	4.73	(1.29–12.08)	

## Appendix

**Table A1:** Comparison of adjusted hazard ratio (aHR) by models in different categorization levels of early discharge (ED). Model 1 is used in the current study.

Outcomes	Model 1, ED ≤ 48 Hours			Model 2, ED ≤ 72 Hours		
	aHR ^1^	(95% CI)	*p*-Value	aHR ^1^	(95% CI)	*p*-Value
Any re-admission	2.39	(1.92–2.97)	<0.001	1.31	(1.11–1.54)	<0.001
Re-admission for C-spine problem	1.72	(SE = 2.13) ^2^	0.660	1.51	(SE = 0.40) ^2^	0.118
Re-admission for second ACDF	3.27	(SE = 18.80) ^2^	0.852	1.98	(SE = 1.00) ^2^	0.178

^1^ Adjusted for age, gender, anemia, chronic peptic ulcer disease, chronic pulmonary disease, depression, diabetes, hypertension, liver disease, and valvular disease. ^2^ The adjusted hazard ratio was estimated based on a multivariate Cox regression model with 1000 repeats of bootstrap samples. Standard error (SE) of adjusted hazard ratio and *p*-value were reported instead of 95% CI. A larger SE means a wider confidence interval and implies less chance that ED associates with the outcome (i.e., C-spine re-admission or re-operation for ACDF).
